# Molecular-evaluated and explainable drug repurposing for COVID-19 using ensemble knowledge graph embedding

**DOI:** 10.1038/s41598-023-30095-z

**Published:** 2023-03-04

**Authors:** Md Kamrul Islam, Diego Amaya-Ramirez, Bernard Maigret, Marie-Dominique Devignes, Sabeur Aridhi, Malika Smaïl-Tabbone

**Affiliations:** Université de Lorraine, CNRS, Inria Nancy Grand-Est, LORIA, 54000 Nancy, France

**Keywords:** Computer science, Computational biology and bioinformatics, Drug discovery

## Abstract

The search for an effective drug is still urgent for COVID-19 as no drug with proven clinical efficacy is available. Finding the new purpose of an approved or investigational drug, known as drug repurposing, has become increasingly popular in recent years. We propose here a new drug repurposing approach for COVID-19, based on knowledge graph (KG) embeddings. Our approach learns “ensemble embeddings” of entities and relations in a COVID-19 centric KG, in order to get a better latent representation of the graph elements. Ensemble KG-embeddings are subsequently used in a deep neural network trained for discovering potential drugs for COVID-19. Compared to related works, we retrieve more in-trial drugs among our top-ranked predictions, thus giving greater confidence in our prediction for out-of-trial drugs. For the first time to our knowledge, molecular docking is then used to evaluate the predictions obtained from drug repurposing using KG embedding. We show that Fosinopril is a potential ligand for the SARS-CoV-2 nsp13 target. We also provide explanations of our predictions thanks to rules extracted from the KG and instanciated by KG-derived explanatory paths. Molecular evaluation and explanatory paths bring reliability to our results and constitute new complementary and reusable methods for assessing KG-based drug repurposing.

## Introduction

The development of a new drug requires a large sum of money (between 2 and 3 billion dollars) and a long time (over 13 years)^1^. The main source of this huge cost is the testing of a large number of drugs in preclinical stages, as well as the substantial percentage of randomised controlled trials (RCTs) that do not show clinical benefits or have toxicity risks^2^. Furthermore, it has a poor success rate, owing to incorrect drug target or response identification^3^. Within this context, exploiting approved and investigational drugs for new indications, a method called “drug repurposing”, can remarkably reduce development cost and time as the clinical profiles of the studied drugs (pharmacokinetic, pharmacodynamic, and toxicity) are already known^4^.

Virtual screening approaches have shown strong impact in drug discovery and repurposing tasks which model the quality of a target protein–drug complex based on docking the drug against the 3D structure of the target protein^5,6^. However, they are known to let false positives through i.e., drugs showing good docking results but not showing any activity in experiments^6^. Analyzing drugs and disease targets from a larger perspective rather than just their structures could reduce false positive rate. In this context, a knowledge graph (KG) is a useful tool which integrates different types of biological data from diverse sources. KGs are multi-relational graphs composed of entities (or nodes) representing several biological concepts (e.g. genes, proteins, drugs) and relations representing physical and biological associations. The integration of diverse data sources enables KG entities to be explored from a larger perspective for capturing complex relationships among diverse biological data and could help to minimize false results in predicted drugs. A KG is represented as a set of triples in the form (*head entity, relation, tail entity*), also called facts^7^. In KGs, the prediction of missing head or tail entities for a triple is known as link prediction^7–9^. Drug repurposing methods based on KGs have emerged as a prominent tool in recent years^10–14^. In a drug repurposing task, a link prediction method is used to compute the probability of a *Treat* relation between a drug and a disease entity.

A KG embedding method actually learns low-dimensional vector representations (or embeddings) of entities and relations while preserving the inherent structure of a KG. The embeddings are then used in downstream tasks on KG, such as link prediction, entity classification, and entity resolution. A link prediction method uses the learned embeddings to predict complex relationships between two entities. A plethora of KG embedding methods exist in the literature, which are broadly categorized into four major categories: translational, semantic matching, random-walk-based, neural network (NN)-based^9^. Methods from the first two categories, translational and semantic matching, became very popular in recent years due to their simplicity and ability to work under the open world assumption (OWA) in knowledge graphs. Translational methods (e.g. TransE^7^, TransH^15^, TransD^16^, TransR^17^, TransF^18^) assume that the sum of head and relation embeddings is nearly equal to the tail embedding if (head, relation, tail) is a fact in the KG. Semantic matching methods (e.g. DistMult^19^, RotatE^20^, QuatE^21^, HolE^22^, Analogy^23^) are based on the semantic similarity between entities and relations in the embedding space. Each embedding method has its own capabilities and limitations for learning embedding of different relations. For example, TransE can not model symmetric relations, DistMult can not model anti-symmetric and inverse relations. Recent works review state-of-art embedding methods^9,24,25^.

In a knowledge graph setting, drug repurposing is formulated as a task of link prediction where the probability of a *Treat* relation is computed from an approved/investigational compound (head) to a disease (tail) i.e. computing the probability of a (*Compound, Treat, Disease*) triple. Based on this formulation, there exist several drug repurposing approaches in the literature. Few of them are generic^26,27^ and the rest are specific to certain diseases^11,28–30^.

**Figure 1:**
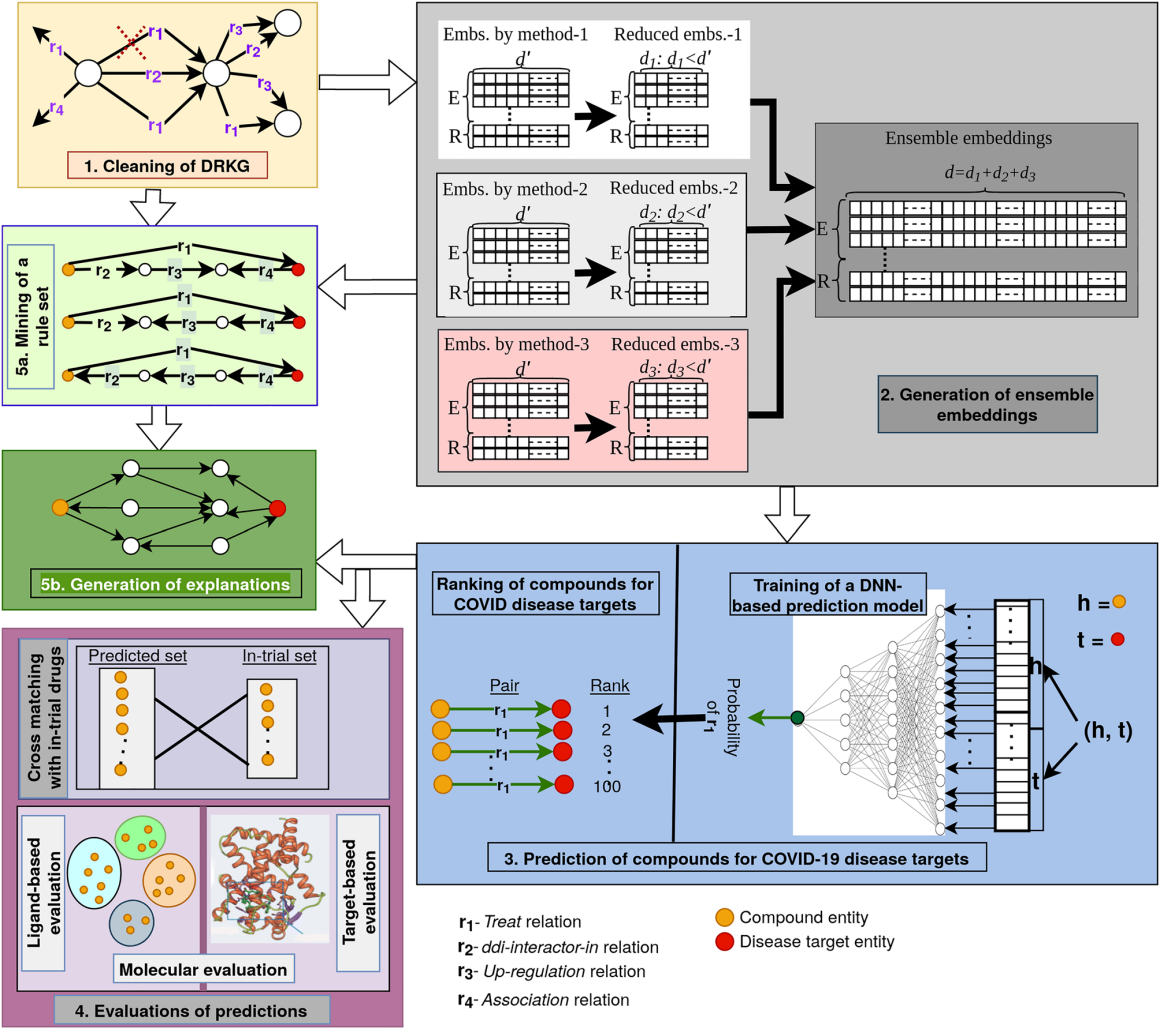
Overall study workflow; the major steps are numbered. Step 1 (yellow box); cleaning a COVID-19 centric drug repurposing knowledge graph (DRKG). Step 2 (gray box): learning high-quality and compact ensemble embeddings. Step 3 (blue box): predicting and ranking potential drugs for COVID-19 disease targets. Step 4 (purple box): evaluation of the top-100 compounds based on cross-matching with in-trial drugs (upper panel) and molecular evaluation of the compounds targeting SARS-CoV-2 nsp13 protein (lower panels). Step 5a (light-green box): learning from DRKG a set of explanation rules. Step 5b (dark green box): extracting explanatory paths instantiating the rules for given (Compounds, Disease) pairs of interest.

The COVID-19 pandemic, caused by severe acute respiratory syndrome coronavirus 2 (SARS-CoV-2), has already costed the lives of almost 6 million people, and it still continues. To the best of our knowledge, no specific drug is available till now against the COVID-19. There exist few drug repurposing studies for the COVID-19^11–14,31^. Most of the approaches use traditional KG embedding methods for learning entity and relation embeddings in COVID-19 centric biological KGs e.g. Drug Repurposing Knowledge Graph (DRKG)^11^ and then use the embeddings in drug repurposing task. Evaluation of predictions against in-trial drugs for COVID-19 is the only way to asses the efficiency of their approaches. In this study, we propose an integrated drug repurposing, evaluation and explanation pipeline for COVID-19 disease. The overall study workflow is illustrated in Fig. 1. We start with collecting and cleaning a COVID-19 centric drug repurposing knowledge graph (DRKG). Then, we propose a novel approach to generate high-quality and compact ensemble embedding of the KG using three traditional embedding methods and the principal component analysis (PCA) method. The embeddings are used to train a deep neural network (DNN)-based prediction model. The trained model is used to predict the probability of all unobserved (*Compound, Treat, COVID-19*) triples where COVID-19 is represented with 27 associated proteins. The triples are then ranked in decreasing order of their probability values and top-100 compounds are predicted as potential compounds for COVID-19. The top-100 predictions are evaluated based on two groups of methods: (1) cross-matching with in-trial drugs for COVID-19 and (2) molecular evaluation based on compound and protein structures. Beside these evaluations, we learn high quality rules from DRKG and provide possible explanations of predictions^32^.

This study differs from state-of-art approaches on three major points. Firstly, other studies depend on a single KG embedding model ignoring the fact that a single model can only learn good embedding of certain relation types^24,25^. On the contrary, we propose an ensemble approach combining multiple embeddings in order to embed different complementary aspects of KG relations. Secondly, existing approaches only asses their predictions against in-trial drugs and do not provide any molecular evaluation of their predictions. We provide molecular evaluation of our predictions by comparison with known ligands of the COVID-19 targets. Finally, whereas KG-derived explanations of predictions are missing in most existing approaches, we provide rule-based explanations extracted from the KG and this contributes to improve the reliability of our predictions. We use the words ‘compound’, ‘drug’ and ‘ligand’ interchangeably throughout the paper.

## Results

In this section, we present our experimental results. We first evaluate embedding models for link prediction task. Then we describe the drug prediction results and different evaluations of our predictions. Finally, we provide explanations for a few predictions.

### The cleaned DRKG

**Figure 2:**
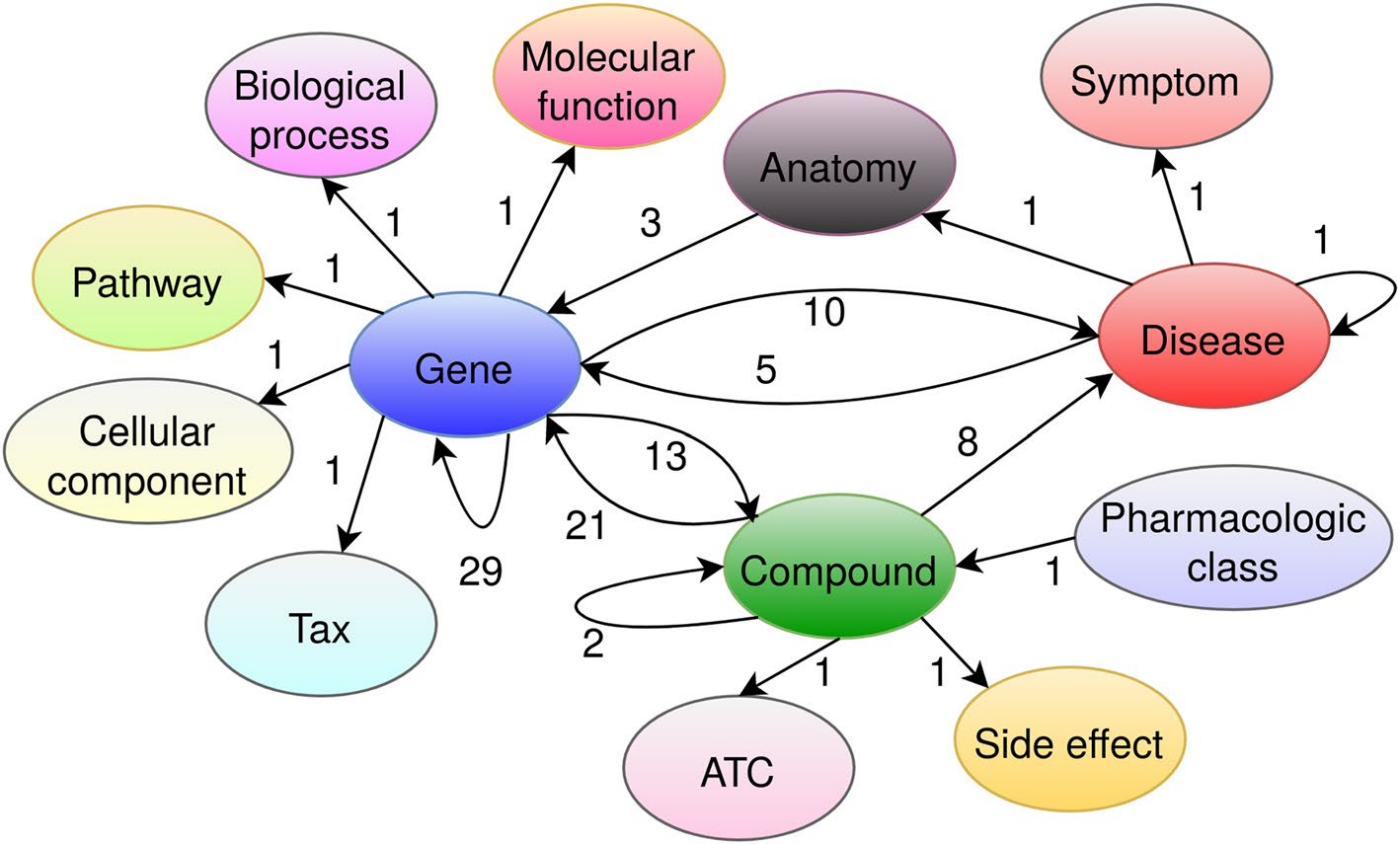
The cleaned DRKG metagraph: the number next to an arrow indicates the number of distinct relations between the corresponding entity types. For example, there are 21 distinct relations from *Compound* to *Gene* entities such as *Binding*, *Down-regulation*, *Up-regulation*, etc.

We first clean the DRKG data^11^ in order to reduce redundant information (see “Materials and methods” section). The cleaned DRKG contains about 98,000 entities of 13 different types: gene, taxonomy (Tax), pathway, biological process, molecular function, cellular component, anatomy, disease, symptom, compound, pharmacologic class, ATC code, side effect (Fig. 2). For our drug repurposing goal, 8103 compound entities, corresponding to approved or in-trial drugs are present in the cleaned DRKG graph. The COVID-19 disease is represented by 27 associated virus proteins (see Supplementary-I, Table S4). The KG contains 102 relation names and 5,813,617 triples. The *Treat* relation exists between compounds and diseases except for COVID-19 disease entities. We provide few statistical information on the cleaned DRKG in Supplementary-I, Fig.S1. We see that more than 65% triples come from two data sources: STRING (a protein interactions knowledge base) and IntAct (a molecular interaction knowledge base). As for the diversity of entity pairs in the triples collected from each data source (see Supplementary-I, Table S1), two data sources focus on only one type of pair: (*Gene, Gene*) for STRING and (*Compound, Gene*) for DGIdb. The five other data sources consider from 2 to 15 types of pairs (for Hetionet).

### Generation of ensemble embeddings

We use complementary classical KG embedding methods to generate embeddings of the cleaned DRKG (see “Materials and methods” section). Beside embedding methods, negative sampling is an important component for learning KG embeddings. We use our recently published the Simple Negative Sampling (SNS) method^8^ for sampling high-quality negative triples. We compared the link prediction performance of SNS with TransE to the frequently used ‘uniform-random’ sampling^7^ with TransE (see Supplementary-I, Fig. S3). As we see approximately 3% improvement in Hit@10 scores, we apply SNS in all embedding methods used here for learning embeddings. To confirm that the cleaning of DRKG does not affect embedding quality, we compare link prediction performance scores in cleaned DRKG to performance scores in original DRKG for three embedding methods: TransE, TransH, and DistMult (see Supplementary-I, Table S3). We see that the performance scores for TransE and TransH do not differ significantly. To avoid the difficulty in tuning the hyper-parameter ‘dimension size’, we first set it to 100 and then use principal component analysis (PCA) to reduce embeddings dimension for each of the three methods used. The embedding dimension size in TransE, DistMult, and TransH reduces to 26, 23, and 25, respectively. The concatenation of these embeddings gives the final ensemble embedding for each entity and relation of dimension size 74. The combination of three embedding methods helps to overcome the weak points of individual embedding methods. The ensemble embeddings of entities and relations are used in downstream drug prediction task.

### Prediction of compounds for COVID-19 disease

We design a DNN-based prediction model to predict the probability that a *Treat* relation exists for a given (*Compound, Disease*) pair. The model is trained with a set of 261,080 training pairs and validated on 5800 test pairs. Using 10-fold cross validation, we find an average mean squared error (MSE) of 0.09 (with standard deviation 0.02) and an average AUC value of 0.96 (with standard deviation 0.01) . The trained model is then used to compute the ranks of the candidate compounds based on their probability values with respect to COVID-19 disease entities. Interestingly, the top-100 predictions do not correspond to a unique but rather to several COVID-19 disease targets. Due to space constraints, we provide only the top-20 predicted compounds in Table 1 and the list of the top-100 ranked compounds in Supplementary-II. We also provide the best disease target for each compound. We see that the ranks of in-trial compounds for COVID-19 are improved noticeably compared to state-of-art approaches (see Table 1). Following aligned studies in literature^11,13^, we consider the top-100 predicted compounds for performing evaluation.

### Evaluations of predictions

To asses the efficiency of our proposed drug repurposing approach, it is important to evaluate our predictions against recommended/in-trial drugs. We perform two types of evaluation for our top-100 ranked compounds: cross-matching with in-trial drugs for COVID-19 and molecular evaluations (only for SARS-CoV-2-nsp13 disease target).

#### Cross-matching with in-trial drugs

When we cross-matched the top-100 compounds with the 31 in-trial compounds for COVID-19 (see Supplementary-I, Table S5), we see that the top-10 ranked compounds are actually in-trial compounds for COVID-19 in Table 1. We find 10/31 in-trial drugs in top-100 predictions in our approach which is remarkable compared to most of state-of-art aligned approaches which also rely on the DRKG knowledge graph^11,13,14,33^ (see last 4 columns in Table 1).

**Table 1:** Top-20 ranked drugs: the in-trial compounds are highlighted in blue texts, – represents unavailability of rank of a drug, * highlights in-trial drugs found only by our approach, the last five columns give the rank or prediction ($\checkmark$) of compounds by different approaches using DRKG.

Compound	Disease target	Our approach	Tex-Graph^13^	TransE-DRKG^11^	ENSIGN^33^	PERM^14^
Dexamethasone	SARS-CoV-2-nsp6	1	1	4	$\checkmark$	$\checkmark$
Methylprednisolone	SARS-CoV-2-nsp6	2	6	16	$\checkmark$	–
Ruxolitinib*	SARS-CoV-2-nsp13	3	–	–	–	–
Colchicine	SARS-CoV-2-nsp6	4	48	8	–	–
Thalidomide	SARS-CoV-2-nsp5_C145A	5	18	–	–	–
Chloroquine	SARS-CoV-2-nsp5_C145A	6	68	–	–	–
Azithromycin	SARS-CoV-2-nsp6	7	13	–	–	–
Losartan	SARS-CoV-2-nsp13	8	41	–	$\checkmark$	–
Baricitinib*	SARS-CoV-2-nsp5_C145A	9	–	–	–	–
Hydroxychloroquine	SARS-CoV-2-nsp5_C145A	10	47	–	–	$\checkmark$
Protirelin (DB09421)	SARS-CoV-2-nsp13	11	–	–	–	–
Telavancin (DB06402)	SARS-CoV-2-nsp13	12	–	–	–	–
Propiomazine (DB00777)	SARS-CoV-2-nsp14	13	–	–	–	–
Hydroxyzine (DB00557)	SARS-CoV-2-nsp13	14	–	–	–	–
Indinavir (DB00224)	SARS-CoV-2-nsp5_C145A	15	–	–	–	–
Nafcillin (DB00607)	SARS-CoV-2-nsp5_C145A	16	–	–	–	–
Bifonazole (DB04794)	SARS-CoV-2-nsp13	17	–	–	–	–
Obeticholic acid (DB05990)	SARS-CoV-2-nsp13	18	–	–	–	–
Meclizine (DB00737)	SARS-CoV-2-nsp13	19	–	–	–	–
Lovastatin (DB00227)	SARS-CoV-2-nsp6	20	–	–	–	–

Results for state-of-the-art drug repurposing approaches are collected from original articles.

The highest number of compounds (40) in top-100 corresponds to the SARS-CoV-2-nsp13 target. Among these 40 compounds 2 are in-trial and 38 are new. We choose to focus our molecular evaluations on these new compounds.

#### Molecular evaluation of compounds for SARS-CoV-2-nsp13

For molecular evaluations, we compare our 38 predicted compounds with 86 compounds known to bind the SARS-CoV-2-nsp13 target (38 from literature and 48 from the PDB database^34^). We provide molecular evaluations based on either ligand or target structures and these are named hereafter ligand-based and target-based evaluations.

##### Ligand-based evaluation

This evaluation consists of clustering the predicted and known ligands based on their structural similarity. We find a total of thirteen clusters. Among these clusters, ten contain both predicted and known ligands and their content is listed in Table 2. We also provide molecular weight (MW) of the maximal common sub-structure (MCS) of ligands in each cluster in the table. The first cluster contains 10 ligands among which 6 are known from the literature and 4 are new predicted compounds. Moreover, this cluster displays the highest MCS, thus revealing a good molecular similarity between all these compounds. Based on the MCS values that display an important decrease between cluster 6 and 7, one could retain all the new compounds from clusters 1 to 6 (i.e. 18 compounds) as potential interesting ligands for the nsp13 target. Interestingly, this set of 18 compounds includes Fosinopril that will also appear in the target-based evaluation.

**Table 2:** Clusters of top-100 predicted and known (from literature and PDB) ligands: the number in ( ) gives integer molecular weight.

No.	Predicted ligands	Known ligands	MW (g/mol) for MCS
1	Fosinopril (563), Griseofulvin (352), Telavancin (1755), Ridaforolimus (990)	Simeprevir (749), Dihydroergotamine (583), Paritaprevir (765), Ergoloid (611), Grazoprevir (766), Ergotamine (581)	120
2	Protirelin (362), Teriparatide (4117), Tiagabine (375)	NUA (193), HR5 (207), VWM (187)	105
3	Binimetinib (441), Niflumic_acid (282), Moxifloxacin (401)	Picrasidine N (490), Irinotecan (586), Netupitant (578), Lumacaftor (452), Bananin (327), Picrasidine M (490), Nilotinib (529), Zelboraf (489)	98
4	Mesoridazine (386), Perphenazine (403), Oxcarbazepine (252), Tizanidine (253)	SSYA10-001 (308), NY7 (194), VVD (197), VW7 (204), S7G (190), UVA (185), N0E (241), NZG (197), JHJ (243), LJA (193), EJQ (222), VXD (198)	93
5	Meclizine (390), Flunarizine (404), Remoxipride (371), Hydroxyzine (374)	Lifitegrast (615), Emend (534), UR7 (203), JOV (227), VWD (200), NX7 (211), VWA (153), UQS (191), UXG (239), VW4 (199)	92
6	Darifenacin(426), Tetrabenazine (317), Oxybutynin (357), Ritodrine (287), Oxymorphone (301), Naloxone (327), Hydrocodone (299), Tofisopam (382)	Tubocurarine (609), Cepharanthine (606), Differin (412), Isorhoeadine (383), Epiexcelsin (414), Enjuvia (350), Homovanillic acid (182), K2P (206), VXG (233), VVY (205), STV (243), VW1 (190)	89
7	Lamotrigine (256)	K34 (152), JG4 (150)	67
8	Carisoprodol (260)	Clavulanic acid (199), Acetylcysteine (163), VWV (221)	59
9	Macitentan (588), Famotidine (337), Eprosartan (424)	Cefoperazone (645), Cefpiramide (612), Risperdal (410), Cordycepin (251), Pritelivir (402), Dpnh (665), VX4 (224)	50
10	Risedronic acid (283), Cinchocaine (343), Bifonazole (310)	RYM (233), NYV (189), VWJ (175), O2A (174), UVJ (203), MUK (199), S7J (191), UJK (203), VWG (188)	50

##### Target-based evaluation

This evaluation consists of performing molecular docking of the 38 predicted and 86 known ligands in the active site of the nsp13 structure using the GOLD software. We provide complete docking results in Supplementary-III to this paper. Table 3 lists the top-20 ligands with respect to molecular docking. Interestingly, 4/38 predicted ligands are present in this list, with docking scores greater than 70. In particular, Fosinopril stands out and is ranked at the second position, with a score (78.86) very similar to the first-ranked ligand Diosmine (79.04), identified as nsp13 ligand by White et al.^35^. The three other predicted ligands: Macitentan, Eprosartan, and Dinoprostone are ranked at position 12 to 14, with scores ranging from 70.76 to 71.76 despite of their varying MW, thus excluding an effect of their size on the number of interactions with the target.

**Table 3:** List of Top-20 best docked ligands ranked according to decreasing Gold Score.

Docking rank	Ligand name	Gold score	MW (g/mol)	Best predicted target	Predicted probability score	Predicted rank	References
1	Diosmin	79.04	608.5	nsp9	0.169	6091	White et al.^35^
***2***	***Fosinopril***	***78.86***	***563.7***	***nsp13***	***0.964***	***29***	***This study***
3	Nilotinib	76.55	529.5	nps6/nsp13	0.860/0.796	101	White et al.^35^
4	Chromone-4c*	76.17	417.4	NA	NA	NA	Perez-Lemus et al.^36^
5	Dpnh	76.04	665.4	nsp9	0.146	6361	White et al.^35^
6	Cromolyn	75.7	468.4	nsp13	0.619	1751	White et al.^35^
7	Picrasidine_N*	74.55	490.5	NA	NA	NA	Vivel-Ananth et al.^37^
8	Picrasidine_M*	74.52	490.5	NA	NA	NA	Vivel-Ananth et al.^37^
9	Dihydroergotamine	74.13	583.7	nsp13	0.796	475	White et al.^35^
10	Ergotamine	72.83	581.7	nsp13	0.805	361	White et al.^35^
11	Simeprevir	72.68	749.9	nsp6/nsp13	0.537/0.139	2076	Gurung ^38^
***12***	***Macitentan***	***71.76***	***588.3***	***nsp13***	***0.95***	***49***	***This study***
***13***	***Eprosartan***	***71.33***	***424.5***	***nsp13***	***0.878***	***71***	***This study***
***14***	***Dinoprostone***	***70.76***	***352.5***	***nsp13***	***0.959***	***38***	***This study***
15	Cefoperazone	70.24	645.7	nsp13	0.599	1816	White et al.^35^
16	Scutellarin*	70.19	462.4	NA	NA	NA	Gurung^38^
17	Irinotecan	70.11	586.7	nsp6/nsp13	0.824/0.796	249	White et al.^35^
18	Paritaprevir	69.61	765.9	orf8	0.099	6990	Gurung^38^
19	Risperdal	68.76	410.5	nsp6/nsp13	0.807/0.807	349	White et al.^35^
20	Ergoloid	68.59	611.7	nsp13	0.584	1888	White et al.^35^

* means that the ligand is not present in DRKG. The results for the ligands from our predictions are highlighted in bolditalic font. The prediction values in DRKG are also indicated with the best disease target and corresponding probability score. When the best target is not nsp13, the second best target and its probability score are indicated only if it is nsp13.

Table 3 also displays the best disease target and probability score of the triples formed between the ligands and COVID-19 disease targets, except for four of them (marked with an asterisk) which are not present in DRKG. When the best disease target is not nsp13, the information for the second best target is provided only if it is nsp13. Surprisingly the best docked ligand Diosmine does not display nsp13 as best or second-best target.

**Figure 3:**
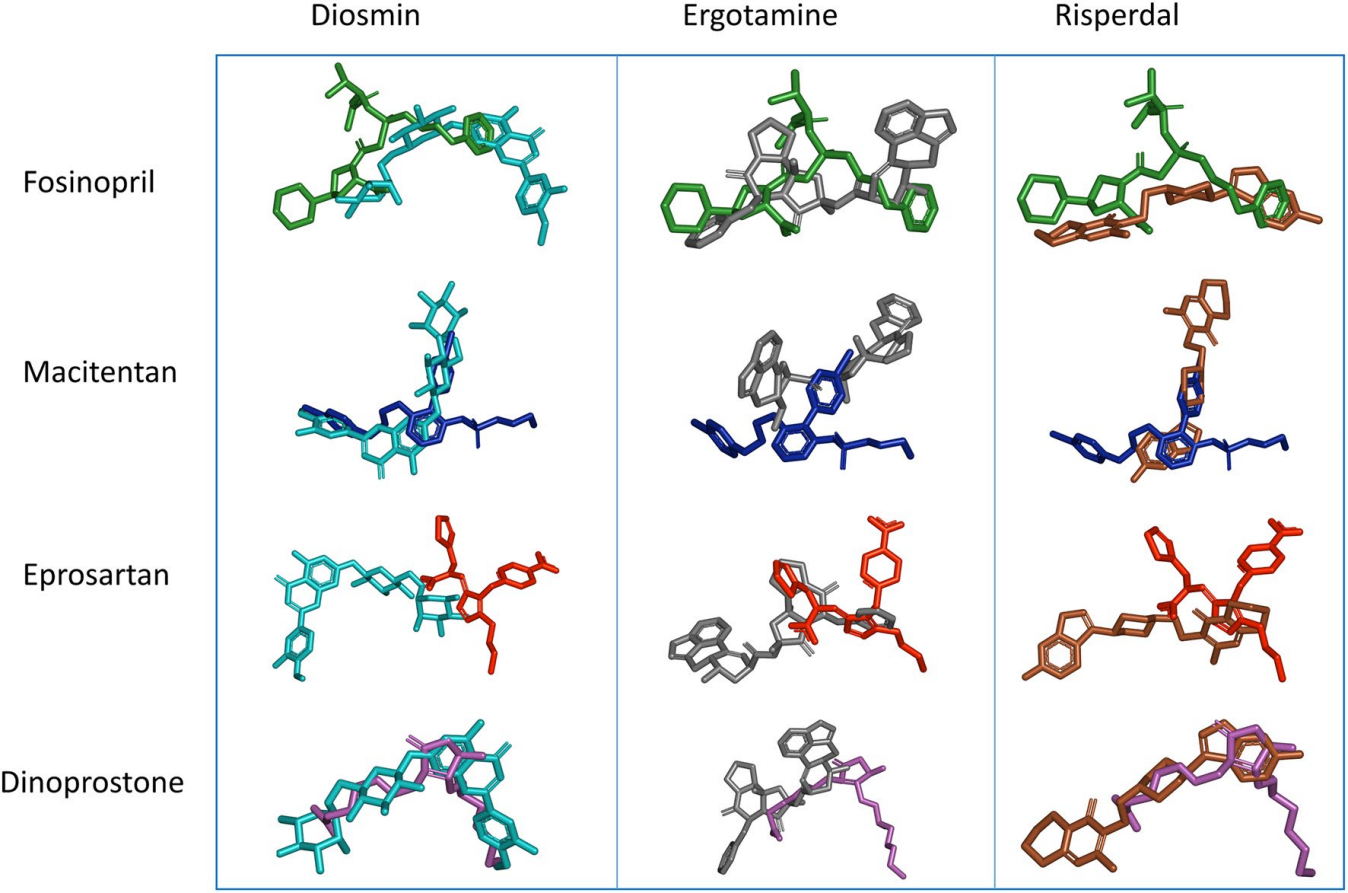
Superposition of binding poses with nsp13 target for Fosinopril (in green), Macitentan (in dark blue), Eprosartan (in red) and Dinoprostone (in magenta) against Diosmin (in cyan), Ergotaminin (in grey) and Risperdal (in brown). Ligands along the vertical axis correspond to the predicted ones, while those along the horizontal axis correspond to the known ones.

We further analyzed our docking results by exploring the interaction maps of our four best-ranked compounds and comparing them with Diosmine (known ligand with the best Gold score), Ergotamine and Risperdal (known ligands with the two highest probability scores with nsp13 in DRKG) to check their binding similarities. Figure 3 shows the superposition of the ligands (4 predicted versus 3 known) extracted from the corresponding docking best poses and Table 4 compares the list of nsp13 residues concerned by interactions with all these ligands.

**Table 4:** List of amino-acid residues in nsp13 structure that interact with the listed ligands (predicted ligands are highlighted in bolditalic font, the others correspond to known ligands).

Ligands	GLY285	GLY287	LYS288	SER289	HIS290	LYS320	GLU375	ARG442	GLY538	GLU540
Diosmin	X	X	X	X	X	X		X	X	X
***Fosinopril***	***X***	***X***	***X***	***X***		***X***	***X***	***X***		***X***
Ergotamine	X		X	X	X	X	X	X	X	
***Eprosartan***			***X***	***X***		***X***				
***Macitentan***					***X***	***X***		***X***		
***Dinoprostone***	***X***	***X***	***X***	***X***	***X***	***X***		***X***		
Risperdal			X	X	X	X	X		X	X

Ligands are ranked as in Table 3. Residue label and position are in bold when they correspond to residues delineating the ATP binding site. All the interaction maps are in Supplementary-I, Table S7 and the full PLIP results can be found in Supplementary-V.

These data show that the predicted compounds share with the known ligands several structural elements and two of them (Fosinopril and Dinoprostone) interact with 6/7 amino-acid residues from the nsp13 active site.

### Explanations of predictions

**Figure 4:**
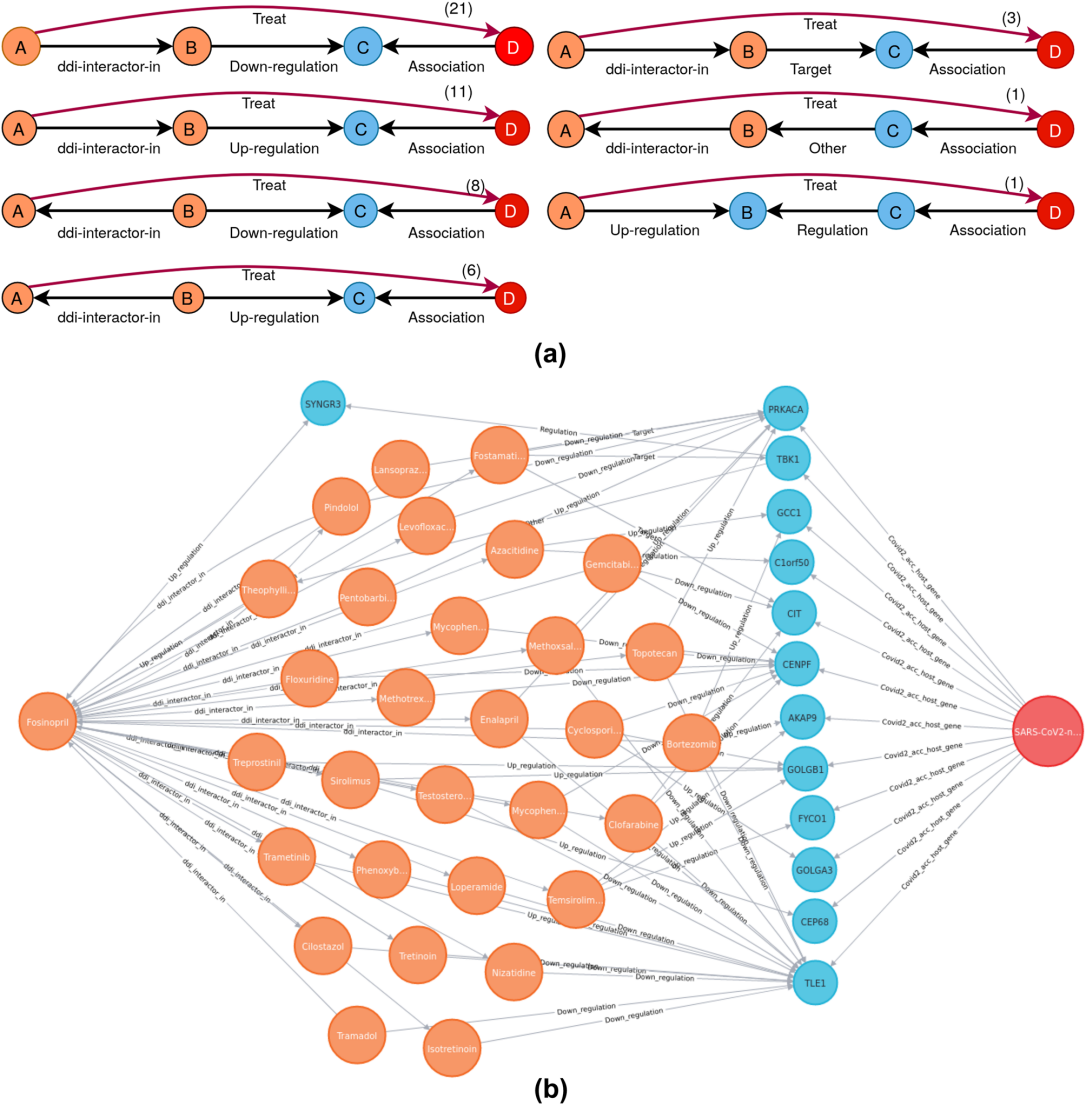
Explanations for the best prediction (with respect to docking rank), Fosinopril-nsp13 pair. Orange, sky and red circles represent compounds, genes and disease targets respectively, black edges represent relations in a rule body and red edge represents the *Treat* relation in a rule head. (**a**) Different rules with at least one path for the pair of interest, () gives the number of paths instantiating a rule. (**b**) Network representation of the paths instantiating the learned rules for the predicted pair (Fosinopril, SARS-CoV-2-nsp13). We see a total of 51 supporting paths between entities of the pair.

For better interpretability and insights about a (*Compound, Treat, Disease*) triple or simply (*Compound, Disease*) pair, we can explore the paths satisfying the learned rule set. As we are interested only in the *Treat* relation between compound and disease target, we learn rules for this relation only. The rule set contains 662 high-quality rules. The explanations for a prediction is generated by instantiating the rules in the DRKG (described in “Materials and methods” section). The complete set of rules is available in Supplementary-IV to this paper. Here, we illustrate (Fig. 4) the paths satisfying the rule set for the best predicted (*Compound, Disease target*) pair in terms of docking rank: (Fosinopril, nsp13). We find 51 paths from the predicted Fosinopril compound to the nsp13 entity satisfying the seven different rules from the rule set. We schematize the rules which instantiate at least one path in Fig. 4a. We see that the first relation in rule body is the interaction between two compounds except in the second last rule and as second relation, one finds different types of regulation between compounds and genes. Among the paths, more than 40% satisfy the first single rule. In Fig. 4b, we provide a graph showing the 51 paths from the predicted Fosinopril compound to the target SARS-CoV-2-nsp13 entity. We see that TLE family member 1, transcriptional corepressor (TLE1) https://www.ncbi.nlm.nih.gov/gene/7088 is an important nsp13-associated gene and is up-regulated or down-regulated by 15 compounds which interact with the Fosinopril compound. This gene is located in cytosol and nucleoplasm, and enables protein binding and transcription corepressor activities^39^. PRKACA (https://www.ncbi.nlm.nih.gov/gene/5566) and CENPF (https://www.ncbi.nlm.nih.gov/gene/1063) are two other genes which have interaction with nearly 10 compounds from DRKG.

The supporting paths for the prediction are useful for experts interested in assessing the biological relevance of each path to the prediction. Explanatory paths provide working hypotheses for assessing the biological relevance of predicted (*Compound, Disease target*) pairs. Here, the hypotheses shown in Fig. 4 suggest that the possible paths from Fosinopril to nsp13 in the KG include more than one step. This apparently contradicts the direct binding hypothesis evaluated above by molecular docking. However one should recall here that such direct binding is a new information not present in the KG and therefore this type of relation could not be retrieved as an explanatory path. In fact, we rather believe that both types of explanations may co-exist, reinforcing the value of a repurposed candidate drugs.

## Discussion

The cross-matching of our top-100 ranked compounds with in-trial compounds for COVID-19 reveals that our approach finds two distinguished in-trial drugs Ruxolitinib and Baricitinib which were not found by any of the aligned approaches in the literature. On January 14, 2022, two new drugs were recommended by World Health Organization (WHO) and Baricitinib was one of the recommendations^40^. WHO recommended Baricitinib strongly for patients with severe or critical COVID-19 as it suppresses the overstimulation of the immune system. For the Ruxolitinib, Iastrebner et al.^41^ found a trend of lower mortality rate with manageable side effects and no direct organ injury among COVID-19 patients taking the drug^41^. To provide more insightful evaluations of our predictions, we compare them to known compounds for COVID-19 disease targets in terms of molecular evaluations results. Concerning the cluster analysis of known and predicted compounds for the SARS-CoV-2-nsp13 disease target, the MCS for the first cluster has the highest value although ligands from this cluster have diversity in their MWs. Fosinopril and Ridaforolimus are two interesting predicted ligands from this cluster as their MWs are close to few known ligands. The findings from cluster analysis are consistent to some extent to docking findings. Ligands from PDB are smaller in size than predicted and literature-based ligands, this fact limits the number of interactions that can be established. In addition, all ligands obtained from the PDB come from a study^42^ using the crystallographic fragment screening technique which is known to be sensitive to low affinity ligands^43^. These could be possible reasons behind the scene that most of the ligands from PDB are ranked lower than ligands from predictions and literature. More than 80% (5 out of 6) known ligands from the first cluster (Table 2) appear in the top-20 of the molecular docking results. From the predicted ligands of this cluster, Fosinopril stands out and is ranked in the second position. As shown in Table 3 (Predicted rank’ column), Fosinopril global rank is 29. However, when we only consider the 40 best compounds that are targeting nsp13, Fosinopril local rank is 15 (see Supplementary-III). Originally, Fosinopril is an angiotensin-converting enzyme (ACE) inhibitor and primarily used for the treatment of mild to moderate hypertension and some types of chronic heart failure^44^. This compound has few rare side effects such as orthostatic hypotension (2.7% cases), sexual dysfunction (1.7% cases), Angina pectoris (1.1–1.6% cases), rash (1–1.4% cases), Angioedema (0.2% cases) and Shock (0.2% cases) (http://sideeffects.embl.de/drugs/3419/). Thus, it appears as a very promising candidate for drug repurposing against COVID-19.

Our docking results are strengthened by the fact that a good portion of the literature-based nsp13 ligands (24 out of 38) are present in DRKG and could be scored by our prediction model for an association with COVID-19 disease entities. Most of them (15 out of 24) have their best or second-best predicted disease entity being nsp13 (Table 3; Supplementary-III). However, we also observe a few conflicts between the findings from prediction model and molecular evaluation. The best-ranked Diosmin in docking results is poorly ranked by our prediction model. Moreover, the best predicted disease target for this ligand is ‘nsp9’ though it was recommended for the nsp13 in the literature^35^. This can be due to a deficit of information about this ligand in DRKG. Indeed, we checked availability of information about this ligand in DRKG in terms of its number of neighbours or degree and we see that its degree is much smaller than the average degree of top-500 compounds in the graph (20 versus  1250). This unfavorable situation for link prediction in KG is confirmed by the relatively low probability score of diosmin (0.162) with its preferred disease target nsp9. The same situation happens to few other conflicts: Dpnh (degree = 302), Lifitegrast (degree = 21), Paritaprevir (degree = 727).

Comparing the binding poses of Diosmin (best docking score, known ligand) and Fosinopril (second-best docking score, predicted ligand), it is noteworthy that considerable segments of both poses have a very similar location (see Fig. 3). Digging deeper into our results, we find that both ligands have in common 7 interactions with the protein which is an outstanding result (see Table 4). Comparing the binding poses and interaction maps between the four best-docked predicted ligands (Fosinopril, Macitentan, Eprosartan and Dinoprostone) and the three best-docked known ligands (Diosmin, Ergotamine and Risperdal), we observe that their 3D position in the nsp13 target largely overlap (Fig. 3). More precisely, Table 4 shows that Dinoprostone and Diosmin share 7 interactions with nsp13 and Fosinopril has not only 7 interactions in common with Diosmin, but also 6 with Ergotamine and 5 with Risperdal. Thus, this analysis of interaction maps shows good consistency between the docked structures of predicted and known ligands, confirming the validity of our predicted nsp13 ligands. These results suggest that our tool is able to make predictions that are structurally consistent with the literature.

Considering now the explanatory paths found for the best predicted compound Fosinopril for the nsp13 disease target (Fig. 4), we detected an interesting path (Fosinopril$\xrightarrow {\text {Up-regulation}}$ SYNGR3 $\xleftarrow {\text {Regulation}}$ TBK1 $\xleftarrow {\text {Covid2}\_\text {acc}\_\text {host}\_\text {gene}}$ nsp13) where a gene up-regulated by the predicted compound is also regulated by a by a gene associated with the disease target nsp13. Synaptogyrin 3 (SYNGR3) (https://www.ncbi.nlm.nih.gov/gene/9143) encodes an integral membrane protein. The exact function of this gene is still not clear, but studies of a similar murine protein reveal that this gene is a synaptic vesicle protein that interacts with the dopamine transporter^45^. TANK binding kinase 1 (TBK1) (https://www.ncbi.nlm.nih.gov/gene/29110) is an important kinase for regulating inflammatory responses to foreign agents^46^. According to the LINC_L1000 connectivity map^47^, Fosinopril up-regulates SYNGR3 with a dysregulation z-score of 4.476 and TBK1 up-regulates SYNRG3 with a z-score of 5.372. Finally, Gordon et al.^48^ provide a protein interaction map where TBK1 interacts with the nsp13 target. This analysis is just an example how to assess biological relevance of different explanations with the help of experts to identify relevant explanations.

Although the current study shows impressive drug repurposing performance for COVID-19, it presents several improvement possibilities. Firstly, the embedding generation approach may generate low-quality embeddings due to data scarcity problem, a common issue for embedding methods. This can affect the subsequent drug repurposing performance by producing false (positive and negative) results as we have seen false negative result for the ‘Diosmin’ compound. Improving the knowledge graph by including more information about compounds, diseases, and other concepts may reduce false results. Secondly, the COVID-19 disease is still evolving and naturally the 2020 built DRKG may lack important information. For example, WHO recommended Sotrovimab (Drug-bank id: DB16355) for COVID-19^40^ which is missing in the current DRKG version. Lacks of important information about the disease and compounds may affect the drug repurposing performance. Inclusion of recent information into the graph could reduce the problem, though this represents a challenging task. Thirdly, choosing the maximum path length, when learning rules and generating explanation(s), is another very challenging task, as high value will generate large number of low-quality rules and low value may miss useful rules. For example, the approach failed to generate any explanation for the predicted (Periciazine, nsp14) pair with maximum path length 3. Lastly, but not the least, both embedding and rule mining methods take high computational time. For example, each of the three embedding method took nearly 7 days to generate embeddings and the rule mining method took nearly 30 h to learn rules. The implementation of both methods in distributed and parallel setting could minimize the problem. Another possible solution is to reduce the size of the KG by cleaning it. Our cleaning process is a step toward this objective.

In conclusion, this study demonstrates how complementary embedding methods can be used to generate high-quality ensemble embeddings of a KG and how to use embeddings for the drug repurposing task. To the best of our knowledge, this study is the first attempt to combine virtual screening methods with KG embedding methods in predicting and evaluating repurposable drugs for COVID-19. Besides the retrieval of many in-trial drugs, both methods show a converging result that ‘Fosinopril’ could be a new potential nsp13 inhibitor. Experimental validation of our predicted ‘Fosinopril’ compound to treat COVID-19 is another potential perspective of this study. The molecular evaluation and explanation(s) of the predictions in this study lead to a trustable conclusion. The rules, learned in this study, could be useful to build query patterns on other similar KG datasets. Though this study focuses on the COVID-19 disease, the drug repurposing framework is generic and could be applied to other diseases for which a KG exists. In this paper, we provide molecular evaluation of predicted compounds for only nsp13. Evaluation of compounds for other COVID-19 disease targets such as nsp6 or nsp5-C145A which appear as preferred targets in our top-20 predicted compounds, is another possible perspective of this study.

## Materials and methods

Hereafter, we describe our methods using the step numbers introduced in Fig. 1.

### Step 1: Cleaning of DRKG

For drug repurposing of COVID-19, we employ DRKG, an Amazon-built COVID-19-centric knowledge graph^11^. DRKG was built from six biological knowledge bases (DrugBank, Hetionet, String, IntAct, DGIdb, GNBR) and three recent COVID-19 related publications^48–50^. It contains biological entities including genes, chemical compounds, diseases, biological processes, side effects, and symptoms. In addition to SARS-CoV-2 related disease entities, DRKG also includes SARS, MERS related disease entities as SARS-CoV-2 has high sequence and infection mode similarity with earlier MERS and SARS-CoV epidemics. The details of DRKG building procedure can be found in the original article^11^. The target COVID-19 disease is represented by different virus proteins which are involved in different stages of SARS-CoV-2 infection in hosts. There are 97,238 entities in the graph. Of these, 24,313 are compounds, 39,220 are genes, 5103 are disease-related entities, and the rest are other types of entities. DRKG contains 102 relation names (see Supplementary-VI, Table S1) and 5,874,261 triples. Some of the relations are actually biologically equivalent, but DRKG considers them differently based on their sources. For example, ‘GNBR::Treat’, ‘DRUGBANK::Treat’ and ‘Hetionet::Treat’ relations represent the treatment relation between compound and disease entities. The same situation happens for ‘Hetionet::Interaction’, ‘STRING::Binding’, ‘INTACT::Direct-interaction’, ‘INTACT::Physical-association’ relations representing the interaction between pair of genes. Because of occurring multiple equivalent relations in the original DRKG, we see redundancies in triples. For example, there are two triples (*Prednisolone, DRUGBANK::Treat, Subacute thyroiditis*) (extracted from the Drugbank) and (*Prednisolone, GNBR::Treat, Subacute thyroiditis*) (extracted from the GNBR) in the DRKG, but they both illustrate the same knowledge that Prednisolone (Drugbank identifier: DB00860) treats the disease Subacute thyroiditis (MESH identifier: D013968). We merge the equivalent relations and remove redundant triples to clean DRKG. We merge the three equivalent treat relations and the four interaction relations in the original DRKG into one ‘Treat’ and one ‘Interaction’ relation respectively in the cleaned DRKG. For simplicity, we use *Treat* to denote the ‘Treat’ relation throughout the paper. In addition, the cleaning of the original DRKG reduces 5 redundant relation names to one, thus removing 60, 644 redundant triples. The meta-graph of the cleaned DRKG is illustrated in Fig. 2.

### Step 2: Generation of ensemble embeddings

For training the embedding models for generating embeddings of entities and relations, DRKG triples are split by 90%–5%–5% to prepare positive train-test-valid sets. We apply the split ratio to relation-wise triples to reduce imbalance among train, test and valid sets. In classical KG embedding methods, the general objective is to give more plausibility scores to positive triples and less scores to negative triples. Let us say $\mathbb {E}$ is the set of entities, $\mathbb {R}$ is the set relations, $\mathbb {S}$ is the set of positive training triples, m is the batch size, $S_m$ is the set of positive triples. KG embedding methods start with randomly initializing embeddings of the entities and relations (see Supplementary-I, Figure S2). The methods then fetch $S_m$ and generate a set of negatives ($S'_m$) using a negative sampling method as negative triples are not readily available. We use our recent Simple Negative Sampling (SNS)^8^ method to generate high-quality negatives. SNS works in three major steps: (1) generate a set of candidate negative triples by corrupting a positive triple (replacing head/tail by other entities) and add recently sampled negative triples, (2) compute sampling probability of each candidate so that this probability is higher when the corrupted entity of the candidate triple is closer to the original entity, (3) select randomly one triple among top-ranked negative triples. $S_m$ and $S'_m$ are combined to form a batch of triples. The embeddings are then improved by minimizing the loss between positives and respective negatives in a batch based on the following objective function (Eq. 1).

1 $$\begin{aligned} \min _{\Theta } \sum _{\forall _{(h,r,t)\in S_m, (h',r,t')\in S'_m}} L(f(h,r,t),f(h',r,t'))+ \lambda reg(\Theta ) \end{aligned}$$

Here, *L* is the loss function, *f* is the scoring function of an embedding method, $\lambda$ is the margin, $reg(\Theta )$ is the regularization term, $(h,r,t)\in S_m$ is a positive triple and $(h',r,t')\in S'_m$ is the corresponding negative triple. We refer to original papers of the methods^7,15,19^ for details about their scoring functions. We use the following pairwise loss function (Eq. 2) due to its suitability in ‘open-world’ assumption.

2 $$\begin{aligned} L(f(h,r,t),f(h',r,t'))=\Big [\lambda - f(h,r,t)+f_r(h',r,t') \Big ]_{+} \end{aligned}$$

Here, $[\cdot ]_+=max(0,.)$ is the hinge function. We refer to Islam et al.^32^ for the architecture of a classical KG embedding method with the SNS negative sampling method.

**Figure 5:**
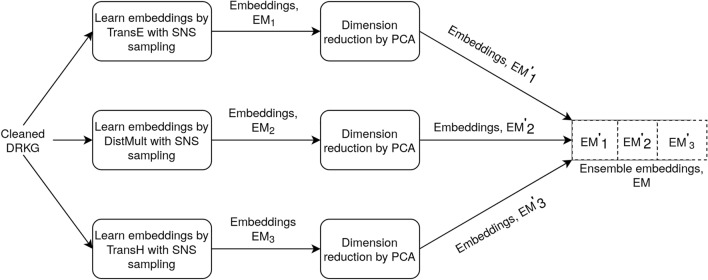
Ensemble embedding learning. Three embeddings of entities and relations are learnt using three embedding methods: TransE, TransH, and DistMult. PCA method is then applied to each of these embeddings for dimensionality reduction. Finally, the three reduced embeddings of each entity or relation are concatenated to generate ensemble embeddings.

There are many KG embedding methods in the literature and every method has its own strong and weak points in learning embeddings for relations with different properties. We follow the analysis conducted by Rossi et al.^25^ on relation properties to select embedding method(s) to learn good quality embeddings of the DRKG. Based on triple statistics, we see that the DRKG contains relations with all properties. For example, the ‘HumGen-HumGen’ relation is symmetric, the ‘Drug-VirGen’ is antisymmetric and the ‘Reaction’ relation is inverse of the relation ‘Catalysis’. Therefore, embeddings of entities and relations in the cleaned DRKG are learned using the three complementary embedding methods (Fig. 5). Indeed, one embedding method TransE (TransH, DistMult) can not handle 1-N and symmetric (symmetric, anti-symmetric and inverse) relations respectively^51^. The triple scoring functions *f* of these embedding methods are available in Supplementary-I, Table S2. The quality of learned embeddings is evaluated in terms of link prediction task. The link prediction performance of an embedding method is defined based on ranks of positive test triples with two widely used metrics: Hit@z, and mean reciprocal rank (MRR)^7,24,51^. The score range of both metrics is 0–1 and higher scores demonstrate better prediction performance. If the rank of a positive test triple *q* is $rank_q$, then the performance metrics are defined in Eqs. (3) and (4).

3 $$\begin{aligned}{} & {} Hit@z=\frac{1}{|\mathbb {D}|}\sum _{q\in \mathbb {D}}hit_q,\; ~hit_q= {\left\{ \begin{array}{ll} 1,&{} if\;{rank_q\le z}\\ 0, &{} \text {otherwise} \end{array}\right. } \end{aligned}$$

4 $$\begin{aligned}{} & {} MRR=\frac{1}{|\mathbb {D}|}\sum _{q\in \mathbb {D}}\frac{1}{rank_q} \end{aligned}$$

As suggested by most of the literature for link prediction in KGs, we consider $z\in \{1,3,10\}$. We re-scale the Hit@z scores from the range 0–1 to 0–100 to facilitate comparisons.

For training embedding methods, one of the important hyper-parameters is the latent space dimension size and finding an optimal value of this parameter requires many experiments which is obviously time consuming and computationally expensive. To avoid the difficulty in tuning this hyper-parameter, we initially set a high value (100) to this parameter. After learning embeddings, we apply the well-known principal component analysis (PCA) method to the embeddings learned by each method in order to reduce their dimension. For embeddings from each method, we keep only the dimensions with high variances by setting a variance ratio threshold of 1% of the total variation.

In a recent study, Chen et al.^52^ argue that embeddings learned by different embedding methods for an object represent different latent features and can be ensembled to one new embedding of the object. As the three embedding methods (TransE, TransH, DistMult) consider different aspects of relations in DRKG, we ensemble the three reduced embeddings to a single new embedding of each entity and relation by simply concatenating them.

### Step 3: Prediction of compounds for COVID-19 disease targets

#### Training of a DNN-based prediction model

For finding repurposable drugs for COVID-19 disease, we design a deep neural network (DNN)-based prediction model which computes the probability of a *Treat* relation for a given (*Compound, Disease*) entity pair. The prediction model is a 4-layer ($148\times 74\times 37\times 1$) multi-perceptron (MLP) architecture with a Rectified Linear Unit (ReLU) activation function. For preparing the positive examples, we consider 52,216 (*Compound, Disease*) entity pairs with the *Treat* relation for the training set and 2900 for the test set. For generating the negative examples, we use our SNS negative sampling method combined with entity-type constraints. For head corruption in a positive pair (*Compound/head, Disease/tail*), we replace the head/Compound entity with other Compound entities from DRKG. As for tail corruption, we replace the tail/Disease entity by other disease entities from DRKG excluding COVID-19 entities. We also check that the negative examples do not correspond to any positive (*Compound, Disease*) pair in DRKG. Then, we apply SNS to select the best four negative pairs (two by corrupting the head, two by corrupting the tail) for each positive pair in training set. Similarly, we select one negative pair for each positive pair in the test set. As a result, the training set contains 52,216 positive and 208,864 negative (*Compound, Disease*) pairs, and the test set contains 2900 positive and an equal number of negative (*Compound, Disease*) pairs. As input to the DNN, each (*Compound, Disease*) pair is represented by the concatenation of the ensemble embeddings of head/Compound and tail/Disease entities, and associated with its positive or negative label. We train the prediction model for a maximum of 4000 epochs with early stopping and 30 epochs patience, Mean Squared Error (MSE) loss, Adam optimizer and a dropout of 0.15 for each layer. The trained model is validated with MSE on the test set. We follow the 10-fold cross validation protocol to validate our drug prediction model. A label-permutation test is performed on our model (see Supplementary-VI for definition). As the resulting p value is below 0.05, we conclude that our classifier exploits in a reliable way the dependency that exists between the sample features and their labels.

#### Ranking of compounds for COVID-19 disease targets

The trained DNN-based prediction model is used to compute probability of an unobserved (*Compound, Treat, Disease*) triple or simply (*Compound, Disease*) pair as the model is trained for the *Treat* relation only. As explained earlier, we are interested to find top-ranked compounds which are expected to treat COVID-19 disease. There are 27 disease entities in the DRKG that represent COVID-19 disease (see Supplementary-I, Table S4) and none of them is linked to any Compound in DRKG. The authors of the DRKG dataset^11^ have integrated in DRKG a set of 8103 FDA-approved or investigational drugs referenced in Drugbank, which have a MW greater than or equal to 250 Da, as candidates for drug repurposing. We use this set of candidate compounds in our *Treat* link prediction experiment. As a result, we have $8103\times 27=218,781$ (*Compound, Disease*) pairs to test. The probability values for all the pairs are computed using the trained prediction model and are ranked in decreasing order. Note that we have 27 (*Compound, Disease*) pairs for each compound and we consider only the best ranked pair among these 27 pairs for each compound. We re-ranked the compounds according to their best rank and obtained a list of 8103 candidates for drug repurposing, with the indication of the best COVID-19 disease target for each of them. Following most of the aligned works^11,13^, the top-100 ranked compounds are proposed as potential compounds to treat COVID-19 disease.

### Step 4: Evaluations of predictions

#### Cross-matching with in-trial drugs

In cross-matching evaluation, the top-100 predicted drugs are cross-matched with the set of in-trial drugs for COVID-19 disease. We use the set of in-trial drugs provided by the DRKG authors in 2020^11^ which consists of 31 compounds. A high number of matches indicates better predictions. This is a simple and quick way of evaluating predictions. To the best of our knowledge, this is the only method used in literature to evaluate a KG embedding-based drug repurposing approach. We follow the hypergeometric law to check the non-randomness of top-100 drug prediction result and we find a very low p value of $4\times 10^{-7}$ (very much below 0.05) which ensures that our finding is far from being a result obtained by chance (see Supplementary-VI).

#### Molecular evaluation

##### Known ligands for the COVID-19 nsp13 protein

As molecular evaluation is an expert and time-consuming task, we perform this type of evaluation only for one disease target: the SARS-CoV-2 nsp13 protein. For collecting known ligands for this disease target, we search both literature and PDB database. We collect 38 known ligands for nsp13 through literature screening^35–38,53,54^. We also extract the ligands for nsp13 found in the PDB entries for nsp13 found in the relevant PDB entries. We obtain 71 entries for nsp13 structures complexed with a ligand in the PDB database and we reduce this number to 48 thanks to a redundancy threshold of Root-Mean-Square Deviation (RMSD) set to 2Å. The list of PDB identifiers (IDs) and corresponding ligands is provided in Supplementary-I, Table S6. This table also contains the PDB ID for the structure of the nsp13 protein without any ligand (PDB:7NIO), also known as apo-nsp13. The 48 PDB ligands are designated hereafter with their capitalized PDB abbreviation. In total, our dataset of known nsp13-ligands contains 86 ligands (48 from PDB and 38 from literature). The 2D structures of all ligands are collected from the PubChem database^55^ in SDF format. We use our 86 known and 38 predicted ligands for two types of molecular evaluation: ligand-based and target-based.

##### Ligand-based evaluation

The ligand-based evaluation is based on the concept of chemical structure similarity that says that is similar ligands or compounds would bind to similar disease targets with almost the same binding affinity and express similar biological responses^5^. This type of evaluation is quick and takes into account the polypharmacological properties of ligands^5^. In ligand-based evaluation, we perform cluster analysis of known and predicted ligands to see how the ligands are grouped based on their similarity. We use the ChemBioServer web application (https://chembioserver.vi-seem.eu/Dendrogram.php) to find clusters of ligands by the Hierarchical clustering method. We select the “Soergel distance” as the distance parameter, “Complete linkage” as the linkage parameter, and different cluster thresholds in {0.6, 0.65, 0.7, 0.75, 0.8, 0.85, 0.9, 0.95} as we find no standard value in the literature for the threshold. A cluster with at least one predicted and at least one known ligand is an interesting cluster to us. Considering the number of interesting clusters, we find 0.9 as a good cluster threshold value. We then compute the Maximum Common Sub-structure (MCS) of ligands in each cluster to estimate the size of the chemical substructure shared by all ligands in a cluster. The higher the molecular weight (MW) of the MCS in a cluster, the higher the sharing degree between cluster members. As one small ligand could be just part of a large ligand, consideration of molecular weight similarity among ligands in a cluster is also an important aspect. It is expected that better predictions will have higher structure sharing and lower difference in MW when compared to known ligands.

##### Target-based evaluation

In target-based evaluation, the binding of ligands to target proteins is assessed from a 3D point of view using a computational approach. Molecular docking is a common computational approach to optimize the process of finding the most favorable 3D binding conformations of the ligand to the target protein^5^. Molecular docking is performed using the GOLD software from Cambridge Crystallographic Data Centre. GOLD stands for Genetic Optimisation for Ligand Docking^56^. The target structure is the apo-nsp13 structure (PDB: 7NIO) lacking any ligand. Based on the literature^35,37,42^, we select the ATP binding site of nsp13 as the binding pocket for molecular docking. The ATP binding site composition in terms of amino-acids (or residues) is as follows: GLU261, ASN265, GLY287, LYS288, SER289, HIS290, LYS320, LYS323, TYR324, ASP374, GLU375, GLN404, ARG442, ARG567. A single structure (the apo-nsp13 one) is used for a target because binding site variations across available PDB entries are weak (checked by RSMD calculation, see Supplementary-I, Table S6). For the ligands, we use the set of known and predicted (from the Top-100 predicted pairs) nsp13 ligands. We extract the 2D structures of these ligands in SDF format from PubChem^55^ and use the Corina tool^57^ (purchased from Molecular Network, GmbH, Nürnberg, Germany; https://mn-am.com/) to transform the 2D SDF format of ligands into 3D structures in MOL2 format. We compute ranks of the ligands in decreasing order of their GOLD docking scores and compare docking ranks with predicted ranks of different ligands. We then explore the interaction maps of few top-ranked predicted and known compounds using PLIP web tool^58^ for deeper analysis and fair comparison.

### Step 5a: Mining of a rule set

Using our neuro-symbolic method^32^, we mine a set of rules from DRKG and use them for generating plausible explanation(s) for the predictions. A rule consists of a rule *Body* and a rule *Head* in the following form:

$$\begin{aligned} Rule: \underbrace{r_1(e_0,e_1)~\wedge ~r_2(e_1,e_2)~\wedge ~\cdots \wedge ~r_n(e_{n-1},e_n)}_\text {Body}\longrightarrow \underbrace{Treat(e_0,e_n)}_\text {Head} \end{aligned}$$

where $e_0, e_1, e_2,\ldots ,e_{n-1},e_n$ are entity variables, $r_1,r_2,\ldots ,r_n$ are relations from DRKG. As we are interested only in the *Treat* relation between compounds and disease targets, the *Treat* relation is the only relation accepted in the rule *Head* and the ($e_0, e_n$) pair is constrained to be a (*Compound, Disease*) pair. Moreover, we limit the size of the rule *Body* to 3 relations only. The rule mining method works in five major steps: (1) sample a subset of triples for the *Treat* relation, (2) extract paths of maximum length 3 from *Compound* to *Disease* entities for one triple, (3) compute scores of the paths based on their entity and relation embedding and rank them, (4) transform top-ranked paths into rules by replacing entities with variables and update the global rule set, (5) iterate steps 2 to 4 for each sampled triple and output the global rule set. We refer to the original article for details about the rule mining method^32^. We use ensemble embeddings of entities and relations and TransE scoring function in the rule mining method. The quality of each rule is evaluated based on a statistical metric named head coverage (HC)^59^ (Eq. 5). HC definition is based on the support of the rule i.e., the number of instances of the rule^59^.

5 $$\begin{aligned} HC(Rule)=\frac{Support(Rule)}{\#(e_0,e_n):Head(Rule)} \end{aligned}$$

The HC metric ranges from 0 to 1. A higher HC metric indicates a better rule.

### Step 5b: Generation of explanations

The mined rules are used to find evidence or investigate drug action mechanisms. We use the mined rule set to generate explanations of predictions. In DRKG, COVID-19 disease entities are connected to host gene entities by only one relation ‘Covid2_acc_host_gene’ pertaining from a study^48^ providing high-confidence interactions between SARS-CoV-2 proteins and human genes. For sake of consistency with the relations occurring in the rule set, we rename this relation as ‘Association’. For each predicted (*Compound, Treat, COVID-19*) triple (COVID-19 represents here one of the 27 SARS-CoV-2 proteins in DRKG), we extract the paths from DRKG starting from the corresponding compound and ending at the corresponding COVID-19 protein, that satisfy at least one of the mined rules. The extracted paths allow us to generate explanations for the predicted relation between the considered compound and COVID-19 target.

## Supplementary Information

Supplementary Information 1.

Supplementary Information 2.

Supplementary Information 3.

Supplementary Information 4.

Supplementary Information 5.

Supplementary Information 6.

## Data Availability

The source code of the proposed framework is available in a GitLab repository (https://gitlab.inria.fr/capsid.public_codes/drug-repurposing-covid19). Data are available in Supplementary materials to this paper. The raw DRKG data were collected from https://github.com/gnn4dr/DRKG.
