# Supplementary material for: Molecular-evaluated and explainable drug repurposing for COVID-19 using ensemble knowledge graph embedding
Source: Sci Rep. 2023 Mar 4;13:3643. doi: 10.1038/s41598-023-30095-z (PMC9985643; doi:10.1038/s41598-023-30095-z)
Supplement: Supplementary file 1 — Supplementary Information 1. [file 41598_2023_30095_MOESM1_ESM.pdf]

Supplementary Information-I

Table S1: Data source-wise entity-pair collection

**Entity-type pair**	**Data Sources**						
↓	Drugbank	GNBR	Hetionet	STRING	IntAct	DGIdb	Bibliography
Gene:Gene		66,722	474,526	1,496,708	254,346		58,629
Compound:Gene	24,801	80, 803	51, 429		1,805	26,290	25,666
Disease:Gene		95,399	27,977				461
Atc:Compound	15,750						
Compound:Compound	1,379,271		6,486				
Compound:Disease	4,968	77,782	1,145				
Gene:Tax		14,663					
Gene:Biological-process			559,504				
Disease:Symptom			3,357				
Anatomy:Disease			3,602				
Disease:Disease			543				
Anatomy:Gene			726,495				
Gene:Molecular-function			97,222				
Compound:Pharma.-class			1,029				
Gene:Cellular-component			73,566				
Gene:Pathway			84,372				
Compound:Side-effect			138,944				

Table S2: Scoring functions of KG embedding methods: h, r and t are head, relation, tail of a triple, embeddings of h, r and t are denoted in bold text, w_r is the normal vector of the hyperplane for r, $diag(r)$ is the diagonal matrix for r, and $||.||_2$ represents l_2 norm.

**Method**	**Embeddings**	**Scoring function,** $f(h, r, t)$
TransE	$\mathbf{h}, \mathbf{t}, \mathbf{r} \in R^d$	$-||\mathbf{h} + \mathbf{r} - \mathbf{t}||_2$
TransH	$\mathbf{h}, \mathbf{t}, \mathbf{r}, w_r \in R^d$	$-||(\mathbf{h} - w_r^T \mathbf{h} w_r) + \mathbf{r} - (\mathbf{t} - w_r^T \mathbf{t} w_r)||_2^2$
DistMult	$\mathbf{h}, \mathbf{t}, \mathbf{r} \in R^d$	$\mathbf{h}^\mathbf{T} diag(r) \mathbf{t}$

Table S3: Link prediction results in raw and cleaned DRKG

**KGE method**	**Original DRKG**				**Cleaned DRKG**			
	MRR	Hit@1	Hit@3	Hit@10	MRR	Hit@1	Hit@3	Hit@10
TransE[7]	0.1670	7.93	20.61	31.66	0.1672	7.99	20.60	31.63
TransH[1]6	0.1602	7.22	20.09	30.50	0.1623	7.54	20.46	30.60
DistMult[1]7	0.1392	4.37	18.63	29.98	0.1417	5.17	19.01	30.03

Table S4: 27 disease entities for the COVID-19 disease

SARS-CoV-2 E	SARS-CoV-2 N	SARS-CoV-2 nsp1
SARS-CoV-2 nsp12	SARS-CoV-2 nsp14	SARS-CoV-2 nsp2
SARS-CoV-2 nsp5	SARS-CoV-2 nsp5 C145A	SARS-CoV-2 nsp7
SARS-CoV-2 nsp9	SARS-CoV-2 orf3a	SARS-CoV-2 orf6
SARS-CoV-2 orf8	SARS-CoV-2 orf9c	SARS-CoV-2 M
SARS-CoV-2 Spike	SARS-CoV-2 nsp10	SARS-CoV-2 nsp13
SARS-CoV-2 nsp15	SARS-CoV-2 nsp4	SARS-CoV-2 nsp11
SARS-CoV-2 nsp6	SARS-CoV-2 nsp8	SARS-CoV-2 orf10
SARS-CoV-2 orf3b	SARS-CoV-2 orf7a	SARS-CoV-2 orf9b

Table S5: 31 clinical trial drugs for the COVID-19 disease

Drug name	Drug name	Drug name	Drug name
Deferoxamine	Piclidenoson	Losartan	Ibuprofen
Favipiravir	Ruxolitinib	Dexamethasone	Thalidomide
Tranexamic acid	Tocilizumab	Sarilumab	Tradipitant
Angiotensin 1-7	Oseltamivir	Baricitinib	Sargramostim
Chloroquine	Anakinra	Mavrilimumab	Azithromycin
Tetrandrine	Bevacizumab	Tofacitinib	Siltuximab
Nivolumab	Nitric Oxide	Colchicine	Remdesivir
Hydroxychloroquine	Eculizumab	Methylprednisolone	

Table S6: Details of PDBs for the SARS-CoV-2 nsp13 protein. PDB-IDs denote identifiers of PDB structures. The ligands from PDBs are given with their PubChem database identifiers (Pubchem-IDs), abbreviated (Abbr.) and IUPAC names. The last column gives Root-Mean-Square Deviation (RMSD) values of PDBs based on ATP binding site.

	**Ligand**			
**PDB-ID**	**Pubchem-ID**	**Abbr.**	**IUPAC name**	**RMSD (Å)**
7nio	-	-	-	0,000
5rli	118569	JFM	N-(2-phenylethyl)methanesulfonamide	0.496
5rlt	225773	UVJ	3-(2-methyl-1H-benzimidazol-1-yl)propanamide	0.513
5rmk	19786643	O2A	N-methyl-1H-indole-7-carboxamide	0.519
5rlj	94347260	VW4	(2S)-2-phenylpropane-1-sulfonamide	0.520
5rmc	2806372	6SU	methyl 3-(methylsulfonylamino)benzoate	0.523
5rm7	836055	N0E	~N-(4-hydroxyphenyl)-3-phenyl-propanamide	0.533
5rmj	978238	JOV	3-chloro-N-(1-hydroxy-2-methylpropan-2-yl)benzamide	0.534
5rl9	63648807	UR7	1-(3-fluoro-4-methylphenyl)methanesulfonamide	0.537
5rl8	4693938	VVG	N-(2-fluorophenyl)ethanesulfonamide	0.541
5rm0	22882465	S7G	~N-[(3 R)-1,2,3,4-tetrahydroquinolin-3-yl]ethanamide	0.543
5rlh	2777223	K2P	2-(trifluoromethoxy)benzoic acid	0.544
5rma	694486	JHJ	N-(4-methoxyphenyl)-N'-pyridin-4-ylurea	0.544
5rm5	51114235	NUA	N-(1-ethyl-1H-pyrazol-4-yl)cyclobutanecarboxamide	0.545
5rlp	8120202	VWA	(1S)-1-(4-fluorophenyl)-N-methylethan-1-amine	0.549
5rlk	71757215	NYV	1-(propan-2-yl)-1H-imidazole-4-sulfonamide	0.550
5rlc	31390	VVM	4-amino-N-phenylbenzene-1-sulfonamide	0.558
5rme	863558	RYM	4-(benzimidazol-1-ylmethyl)benzenecarbonitrile	0.561
5rlo	43539927	UQS	N-[(2-fluorophenyl)methyl]-1H-pyrazol-4-amine	0.563
5rm6	33381566	HR5	~N-(cyclobutylmethyl)-1,5-dimethyl-pyrazole-4-carboxamide	0.570
5rm2	330448	UXG	1-(diphenylmethyl)azetidin-3-ol	0.573
5rmg	91650	MUK	4,6-dimethyl- N-phenyl-pyrimidin-2-amine	0.574
5rmm	155387694	VXG	(3S,4R)-1-acetyl-4-phenylpyrrolidine-3-carboxylic acid	0.580
5rm3	68423044	S7J	2-(trifluoromethyl)pyrimidine-5-carboxamide	0.582
5rlz	124505295	VWM	(3R)-1-acetyl-3-hydroxypiperidine-3-carboxylic acid	0.584
5rlm	71757930	VW7	N-(8-methyl-1,2,3,4-tetrahydroquinolin-5-yl)acetamide	0.588
5rm9	934518	EJQ	~N-(4-fluorophenyl)-2-pyrrolidin-1-yl-ethanamide	0.591
5rld	910270	VVY	2-phenoxy-1-(pyrrolidin-1-yl)ethan-1-one	0.599
5rly	57116692	K34	5-(1,3-thiazol-2-yl)-1H-1,2,4-triazole	0.603
7nng	43363487	UJK	1-(2-methylphenyl)-1,2,3-triazole-4-carboxylic acid	0.616
5rlr	12684717	VWD	(1R)-2-(methylsulfonyl)-1-phenylethan-1-ol	0.623
5rl6	828139	LJA	N-[3-(carbamoylamino)phenyl]acetamide	0.632
5rlg	43082989	VW1	(2S)-2-(4-cyanophenoxy)propanamide	0.642
5rmd	961974	VWY	N-ethyl-4-[(methylsulfonyl)amino]benzamide	0.656
5rm1	2760997	RY4	N-[4-(aminomethyl)phenyl]methanesulfonamide	0.699
5rlu	4291023	JG4	2-(thiophen-2-yl)-1H-imidazole	1.184
5rlf	16226828	NY7	N-(2-methoxy-5-methylphenyl)glycinamide	1.205
5rln	45792228	NZG	3-(acetylamino)-4-fluorobenzoic acid	1.230
5rmh	96462663	VX4	[(4S)-4-methylazepan-1-yl](1,3-thiazol-4-yl)methanone	1.256
5rmf	588246	NX7	(2,6-difluorophenyl)(pyrrolidin-1-yl)methanone	1.273
5rlv	16653131	VWJ	N-(propan-2-yl)-1H-benzimidazol-2-amine	1.273
5rlb	53516552	VVJ	N-cycloheptyl-N-methylmethanesulfonamide	1.283
5rl7	19261749	VVD	5-(acetylamino)-2-fluorobenzoic acid	1.288
5rmb	2763377	VWV	ethyl (1,1-dioxo-1lambda 6 ,4-thiazinan-4-yl)acetate	1.289
5rls	148031	VWG	N-hydroxyquinoline-2-carboxamide	1.304
5rlq	14196351	UVA	N-methyl-2-(methylsulfonyl)aniline	1.313
5rml	2517697	VXD	N-(3-chloro-2-methylphenyl)glycinamide	1.316
5rlw	1530178	S9S	~N-[2-(4-fluorophenyl)ethyl]methanesulfonamide	1.341
5rmi	686921	STV	~N-(1,3-benzodioxol-5-ylmethyl)ethanesulfonamide	1.349
			**Average RMSD**	**0.777**

Table S7: List of residues in nsp13 structure interacting with the listed ligands (predicted ligands are highlighted in light cyan, the others correspond to known ligands). Ligands are ranked as in Table 3. Residue label and position are in bold when they correspond to residues delineating the ATP binding site. One-letter residue names are used.

Ligands	**E261**	**N265**	P283	G285	**G287**	**K288**	**S289**	**H290**	A313	A316	**K320**	**K323**	**E375**	D401	**Q404**	L438	G439	T440	**R442**	R443	Q537	G538	E540	K569
Diosmin	X		X	X	X	X	X	X			X						X	X	X	X		X	X	
Fosinopril				X	X	X	X		X	X	X		X						X				X	
Ergotamine				X		X	X	X			X		X						X	X		X		X
Eprosartan						X	X		X		X			X	X									
Macitentan								X			X	X							X					
Dinoprostone		X		X	X	X	X	X			X					X			X					
Risperdal						X	X	X			X		X								X	X	X	

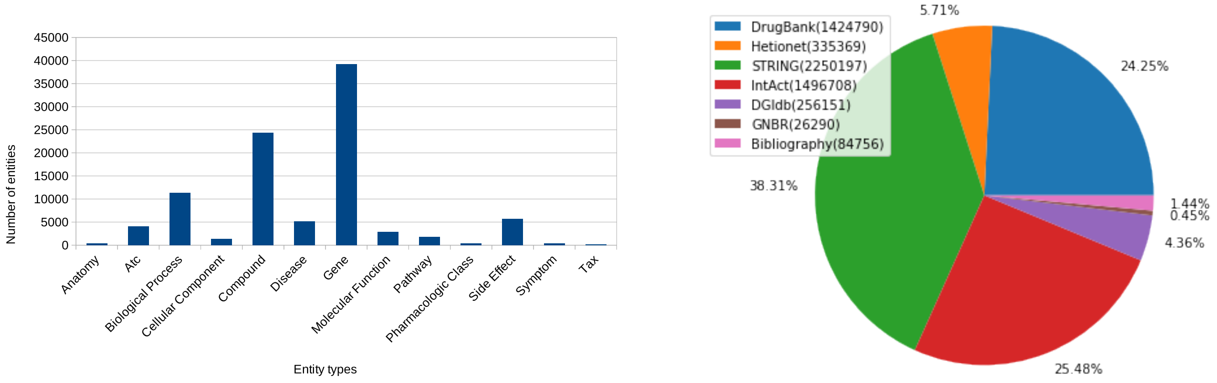

(a) Type-wise entity count

(b) Source-wise triple count

Figure S1: DRKG statistics: (a) Approximately 75% entities are either genes or compounds, (b) Approximately 65% of triples come from two data-source STRING and IntAct. The rest triples come from other remaining five data-sources.

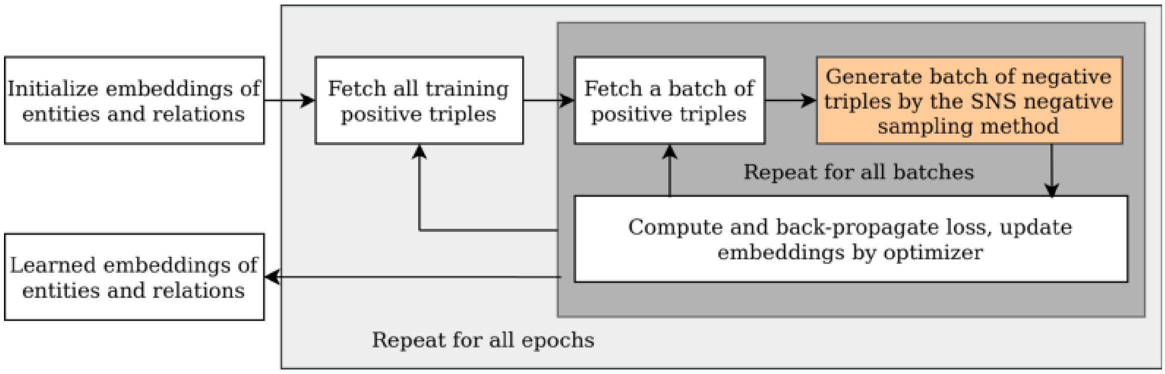

Figure S2: Architecture of a classical KG embedding model with SNS sampling[8].

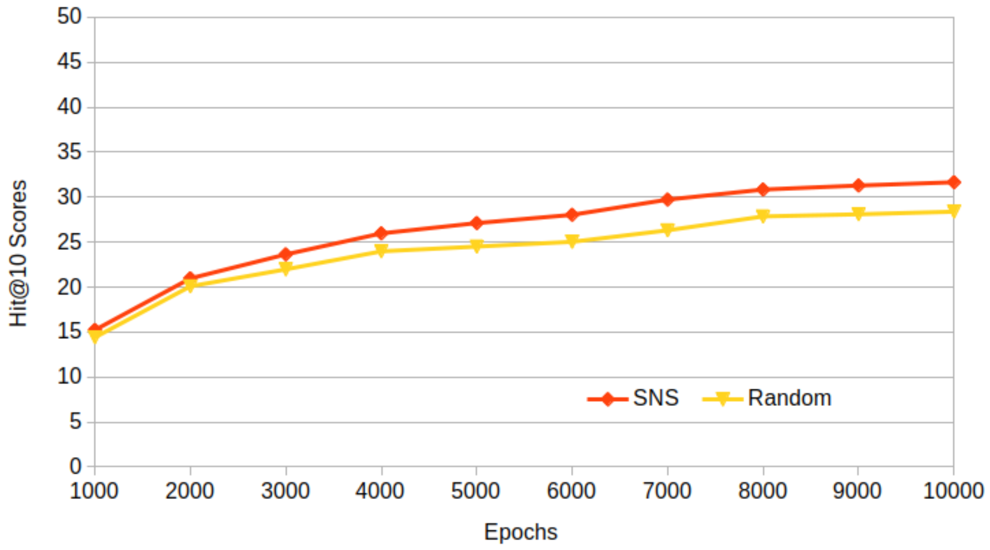

Figure S3: Hit@10 scores for SNS and Random sampling with TransE method for different epochs.
