# Supplementary material for: Molecular-evaluated and explainable drug repurposing for COVID-19 using ensemble knowledge graph embedding
Source: Sci Rep. 2023 Mar 4;13:3643. doi: 10.1038/s41598-023-30095-z (PMC9985643; doi:10.1038/s41598-023-30095-z)
Supplement: Supplementary file 6 — Supplementary Information 6. [file 41598_2023_30095_MOESM6_ESM.pdf]

Supplementary-VI

1 Assesing the significance of the cross-matching of the prediction results with in-trial drugs

Our study matched the Top-100 (not only the Top-20) drugs ranked by our model with in-trial drugs and we found only 10 in-trial drugs which are displayed in the Top-20 drugs listed in Table 1. The complete Top-100 is displayed in Supplementary II as indicated in the paper.
The probability to obtain at random X in-trial drugs in the Top-K drugs ranked by our model, from a set of N=8103 drugs in total, including Y=31 in-trial drugs is given by the following hypergeometric law.

$$p(X) = \frac{\binom{Y}{X} \times \binom{N-K}{K-X}}{\binom{N}{K}}$$

Here, $\binom{Y}{X}$ is the binomial coefficient of Y and X. For K= 100 and X=10 we obtain a p-value of 1.81×10^{-12} , that must be corrected for multiple testing. Indeed each score is predicted 27 times (one for each COVID-19 target) for the 8103 drugs, leading to a total number of tests of 218,781, rounded as 2.2×10^5. Multiplying the p-value by this number yields 3.98×10^{-7}.

2 Permutation test for assesing the significance of our drug prediction model

For permutation test we follow the article:
Ojala & Garriga (2010). Permutation Tests for Studying Classifier Performance. Journal of Machine Learning Research, vol 11, pp. 1833-1863.

At first, we prepare 10 training/test sets from the original dataset. Then, for each split (D):

1. compute the AUC on the test set of the prediction model trained on the training set
2. randomly shuffle labels of training and test independently for 100 times to get 100 permuted sets $\hat{D}$ of D
3. For each $D' \in \hat{D}$, train the prediction model using permuted training set and compute AUC on the permuted test set
4. Compute the p-value on AUC values computed on $\hat{D}$ using the formula:

$$p - value = \frac{|\{D' \in \hat{D} : AUC(f(D')) > AUC(f(D))\}| + 1}{|\hat{D}| + 1}$$

   Here, f is the prediction model. And we see from this formula that for $|\hat{D}| = 100$, the best and worst possible p-values are 0.01 and 1.00 respectively.

In the following figure, we visualize the distribution of AUC values for one split (representative of the distributions for other splits). Finally, the average p-value is computed over the 10 splits yielding p=0.01 (Std = 0).

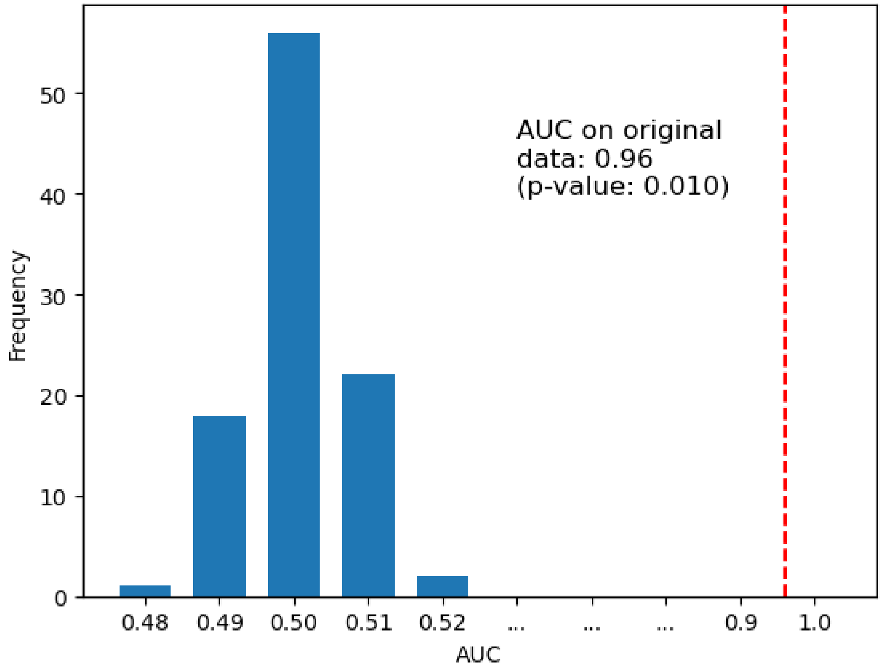

Figure S1: Distribution of AUC values for 100 permutations in a single split: The red line represents the AUC computed on the original dataset

3 Details of relations in the cleaned DRKG

In the original DRKG knowledge graph, relation names are written in the form 'Data-source::Relation-name::Head type:Tail type'. In this table, we simplify all relations by only keeping 'Relation-name'. In case of a relation occurring multiple times(such as 'Other' or 'Association'), we append a number to such relations to differentiate them. Table S1 gives the list of simplified relations with a short description.

Table S1: Relations in the cleaned DRKG graph. The first column denotes the relation name as provided in the data source (last column). The second column gives a short description, the third and fourth columns denote the head and tail entity types respectively. Relations are sorted according to their data source.

Relation	Short description	Head entity type	Tail entity type	Source
HumGen-HumGen	Physical interaction among host proteins	Gene	Gene	Biorxiv
VirGen-HumGen	Physical interaction between virus and host proteins	Gene	Gene	Biorxiv
Drug-VirGen	Interaction between drug and virus proteins	Compound	Gene	Biorxiv
Drug-HumGen	Interaction between drug and host proteins	Compound	Gene	Biorxiv
Covid2_acc_host_gene	High-confidence SARS-CoV-2 and host protein interaction	Disease	Gene	Biorxiv
Coronavirus_ass_host_gene	Coronavirus-host association (experimental evidence)	Disease	Gene	Biorxiv
Inhibitor	Binding, decrease expression	Gene	Compound	DGIDB
Antagonist	Blocking or dampening agonist-mediated responses	Gene	Compound	DGIDB
Other-1	Interaction that doesn't belong to other interactions in DGIDB	Gene	Compound	DGIDB
Agonist	Binding, activating the receptor	Gene	Compound	DGIDB
Binder	Physical binding	Gene	Compound	DGIDB
Modulator	Regulating target without involving in any direct binding to the target.	Gene	Compound	DGIDB
Blocker	Blocking interaction	Gene	Compound	DGIDB
Channel-Blocker	Channel blocking interaction	Gene	Compound	DGIDB
Antibody	Antibody-target binding interaction	Gene	Compound	DGIDB
Positive-Modulator	Increasing activity of the target enzyme	Gene	Compound	DGIDB
Allosteric-Modulator	Modulator interaction where drugs exert their effects on targets via a different binding site than the orthosteric ligand site	Gene	Compound	DGIDB
Activator	Activating biological responses from targets	Gene	Compound	DGIDB
Partial-Agonist	Eliciting reduced amplitude functional responses at target receptors	Gene	Compound	DGIDB
x-atc	Anatomical Therapeutic Chemical (ATC) code	Compound	Atc	DrugBank
ddi-interactor-in	Interaction among drugs	Compound	Compound	DrugBank
Target	Drug-disease target interaction	Compound	Gene	DrugBank
Enzyme	Drug-enzyme interaction	Compound	Gene	DrugBank
Carrier		Compound	Gene	DrugBank
Treat	Treatment interaction	Compound	Disease	Merged
E	Affects expression (neutral)	Compound	Gene	GNBR
A+	Agonism, activation	Compound	Gene	GNBR
N	Inhibits	Compound	Gene	GNBR
K	Metabolism, pharmacokinetics	Compound	Gene	GNBR
A-	Antagonism, blocking	Compound	Gene	GNBR
E+	Increasing expression	Compound	Gene	GNBR
B	Binding, ligand (esp. receptors)	Compound	Gene	GNBR
E-	Decrease expression	Compound	Gene	GNBR
O	Transport, channels	Compound	Gene	GNBR
Z	Enzyme activity	Compound	Gene	GNBR
C	Inhibiting cell growth	Compound	Disease	GNBR
Sa	Side effect/adverse event	Compound	Disease	GNBR
Pa	Alleviating, reducing	Compound	Disease	GNBR
Mp	Biomarkers (of disease progression)	Compound	Disease	GNBR
Pr	Prevents, suppresses	Compound	Disease	GNBR
J	Role in disease pathogenesis	Compound	Disease	GNBR
L	Improper regulation linked to disease	Gene	Disease	GNBR
U	Causal mutations	Gene	Disease	GNBR
Y	Polymorphisms alter risk	Gene	Disease	GNBR
J	Role in pathogenesis	Gene	Disease	GNBR
Te	Possible therapeutic effect	Gene	Disease	GNBR
Md	Biomarkers (diagnostic)	Gene	Disease	GNBR
G	Promotes progression	Gene	Disease	GNBR
D	Drug targets	Gene	Disease	GNBR
X	Overexpression in disease	Gene	Disease	GNBR
Ud	Mutations affecting disease course	Gene	Disease	GNBR
V+	Activates, stimulates	Gene	Gene	GNBR
Q	Production by cell population	Gene	Gene	GNBR
Rg	Regulation	Gene	Gene	GNBR

B	Binding, ligand (esp. receptors)	Gene	Gene	GNBR
I	Signaling pathway	Gene	Gene	GNBR
E+	Increasing expression	Gene	Gene	GNBR
H	Same protein or complex	Gene	Gene	GNBR
W	Enhancing response	Gene	Gene	GNBR
E	Affecting expression (neutral)	Gene	Gene	GNBR
In_tax	Gene taxonomy	Gene	Tax	GNBR
GpBP	Participation	Gene	Biological-Process	Hetionet
GiG	Interaction	Gene	Gene	Merged
CrC	Resembling	Compound	Compound	Hetionet
DdG	Down-regulation	Disease	Gene	Hetionet
DpS	Presence	Disease	Symptom	Hetionet
DlA	Localization	Disease	Anatomy	Hetionet
CbG	Binding	Compound	Gene	Hetionet
Up-regulation (CuG)	Up-regulation	Compound	Gene	Hetionet
DrD	Resembling	Disease	Disease	Hetionet
Association (DaG)	Association	Disease	Gene	Hetionet
CpD	Palliation	Compound	Disease	Hetionet
AdG	Down-regulation	Anatomy	Gene	Hetionet
AuG	Up-regulation	Anatomy	Gene	Hetionet
GcG	Covariation	Gene	Gene	Hetionet
GpMF	Participation	Gene	Molecular-Function	Hetionet
PCiC	Inclusion	Pharmacologic-Class	Compound	Hetionet
GpCC	Participation	Gene	Cellular-Component	Hetionet
Regulation (Gr>G)	Regulation	Gene	Gene	Hetionet
Down-regulation (CdG)	Down-regulation	Compound	Gene	Hetionet
DuG	Up-regulation	Disease	Gene	Hetionet
GpPW	Participation	Gene	Pathway	Hetionet
CcSE	Cause	Compound	Side-Effect	Hetionet
AeG	Expression	Anatomy	Gene	Hetionet
Physical-Association	Interaction between molecules within the same physical complex	Compound	Gene	INTACT
Direct-Interaction	Interaction between direct in-contact molecules	Compound	Gene	INTACT
Association-1	Interaction between participating molecules that form one	Compound	Gene	INTACT
Association-2	Interaction between participating molecules that form one	Gene	Gene	INTACT
Colocalization	Interaction between molecules that are affiliated with the same cellular structure	Gene	Gene	INTACT
Dephosphorylation	Relation among genes participating in dephosphorylation reaction	Gene	Gene	INTACT
Cleavage	Gene-gene relation as cleavage reaction occurs	Gene	Gene	INTACT
Phosphorylation	Gene-gene relation as phosphorylation reaction occurs in signal transduction	Gene	Gene	INTACT
ADP-Ribosylation	Relation among genes participating in ADP-Ribosylation reaction	Gene	Gene	INTACT
Ubiquitination	Relation between Ubiquitin-protein and substrate protein as ubiquitination reaction occurs	Gene	Gene	INTACT
Protein-Cleavage	Gene-gene relation as protein cleavage reaction occurs	Gene	Gene	INTACT
Reaction	Gene-gene relation as other types of reaction occur	Gene	Gene	STRING
PTMOD	Gene-gene relation because of post-translational modification	Gene	Gene	STRING
Catalysis	Gene-gene relation as catalysis occurs	Gene	Gene	STRING
Activation	A gene activates another gene	Gene	Gene	STRING
Inhibition	A gene prevents another gene expression at the post-transcriptional level	Gene	Gene	STRING
Other-2	Other type of gene-gene relation	Gene	Gene	STRING
Expression	Expression among genes	Gene	Gene	STRING
